# Technology Use of Older Adults and Caregivers: Discoveries and Opportunities for Improvement

**DOI:** 10.1093/geroni/igaa057.2999

**Published:** 2020-12-16

**Authors:** Justine Sefcik, Jina Huh-Yoo

## Abstract

Innovative technology can improve the lives of older adults, including those diagnosed with dementia, and their caregivers. Yet a lack of careful attention to preferences and needs of end-users and continuous updates to resources could leave consumers without a valuable user experience. This symposium will cover exemplar cases of innovative technologies, available resources, and current research. The first presentation will discuss virtual reality used among older adults with dementia and the opportunities to further explore it’s use as an intervention. The second presentation will share the process of seeking stakeholders’ preferences in the design specifications for a socially assistive robot and how the perspectives shaped the development of the Quori robot. The third presentation will focus on detailing the Information Quality Framework for Online Dementia Care Resources. The fourth presentation will discuss the implications of a systematic review that revealed researchers are reporting on all older adults within a category of 65 and older, thus failing to present variance among different older age cohorts. These presentations will all conclude with a discussion on opportunities for improvement in the respective areas.

